# Relation between Vitamin D level and severity, symptomatology and cognitive dysfunction of Major Depressive Disorder - A sample of Egyptian patients

**DOI:** 10.1192/j.eurpsy.2022.1419

**Published:** 2022-09-01

**Authors:** S. Elsessy, M. Khalil

**Affiliations:** Kasr Alainy Hospitals, Cairo University, Psychiatry And Addiction, cairo, Egypt

## Abstract

**Introduction:**

Vitamin D helps in the regulation of neurotransmission and neuroprotection. Therefore, vitamin D deficiency might lead to inactivated receptors and may result in depression.

**Objectives:**

The study assessed the relation between serum level of vitamin D and severity, symptomatology and cognitive dysfunction of Major Depressive Disorder (MDD) in a sample of Egyptian patients.

**Methods:**

Serum levels of 25-hydroxy vitamin D were measured with electro-chemiluminescence binding assay technique in 75 patients with major depressive disorder. Patients were recruited from Psychiatry and Addiction Hospital, Kasr Al Ainy outpatient clinic. Patients were subjected to the Structured Clinical Interview for DSM-IV Axis I Disorders(SCID), Hamilton depression scale (HAM-D), Mini-mental status examination (MMSE), Wechsler memory subtests (story A and paired associate learning test (PALT) ), Benton visual retention test (BVRT) and Trail B test.

**Results:**

94.6% of patients had vitamin D deficiency. There was no significant correlation between levels of vitamin D and severity of depression according to HAM-D. Regarding symptoms of depression, there was a statistically significant difference between levels of vitamin D, being more deficient with decreased concentration, decreased libido and menstrual disturbances. There was no statistically significant correlation between level of vitamin D and cognitive functions tests.

**Conclusions:**

Major depressive disorder was associated with vitamin D deficiency but no statistical significant correlation could be established neither between levels of vitamin D and severity of depression nor between levels of vitamin D and cognitive dysfunction. Vitamin D level was statistically correlated with decreased concentration, decreased libido and menstrual disturbances.

**Disclosure:**

No significant relationships.

